# Sensorimotor Integration by Targeted Priming in Muscles with Electromyography-Driven Electro-vibro-feedback in Robot-Assisted Wrist/Hand Rehabilitation after Stroke

**DOI:** 10.34133/cbsystems.0507

**Published:** 2026-01-27

**Authors:** Legeng Lin, Yanhuan Huang, Wanyi Qing, Man-Ting Kuet, Hengtian Zhao, Fuqiang Ye, Wei Rong, Waiming Li, Xiaoling Hu

**Affiliations:** ^1^ Department of Biomedical Engineering, The Hong Kong Polytechnic University, Hong Kong SAR 999077, China.; ^2^ Research Institute for Smart Ageing (RISA), The Hong Kong Polytechnic University, Hong Kong SAR 999077, China.

## Abstract

Restoring precise muscular control in the poststroke wrist/hand (W/H) demands sensorimotor integration to correct compensatory neuroplasticity. However, current rehabilitation robots inadequately modulate ascending somatosensory pathways from specific muscles. This study developed an electromyography (EMG)-driven soft robot with electro-vibro-feedback (EVF-robot) for targeted somatosensory priming in W/H muscles. This system integrates (a) focal vibratory stimulation and neuromuscular electrical stimulation for recruiting the somatosensory pathways of the targeted W/H flexors and extensors; (b) an EMG-driven control algorithm for strengthening the voluntary motor control of a driving muscle; and (c) robot assistance to achieve coordinated joint extension and flexion. In a single-arm trial with 20 sessions, 15 chronic stroke participants assisted by the system achieved significant improvements in voluntary W/H behavioral control, somatosensory feedback, and intermuscular coordination in the paretic upper limb (*P* < 0.05). During their W/H extension, the cortical peaks of corticomuscular coherence shifted contralaterally for W/H extensors, and the ascending corticomuscular coherence from W/H flexors increased (*P* < 0.05). These improvements persisted at the 3-month follow-up. The findings provide preliminary evidence that sensorimotor integration training with the EMG-driven EVF-robot may modulate compensatory neuroplasticity and facilitate improvements in coordinated motor control of the distal joints in individuals with chronic stroke.

## Introduction

People with chronic stroke progressively develop compensatory movements in the impaired upper limb (UL) to achieve daily tasks [1,2]. For example, functional compensation in the proximal shoulder/elbow (S/E) joints for distal wrist/hand (W/H) dysfunction is highly prevalent [1,2]. Although compensatory strategies are widely adopted in early poststroke rehabilitation to facilitate hospital discharge, they can lead to muscular learned disuse in the W/H, which is associated with a limited range of motion, abnormal interjoint motions, and pain, thereby limiting the potential of motor recovery [2–4]. Studies reveal that compensation arises from maladaptive neuroplasticity at both the central nervous system and peripheral muscular levels during rehabilitation, e.g., hyperexcitability of the unaffected hemisphere [5,6], impaired inhibition in the spinal cord [7], and discoordination in agonist–antagonist and proximal–distal muscles [3,8,9].

**Fig. 1.**
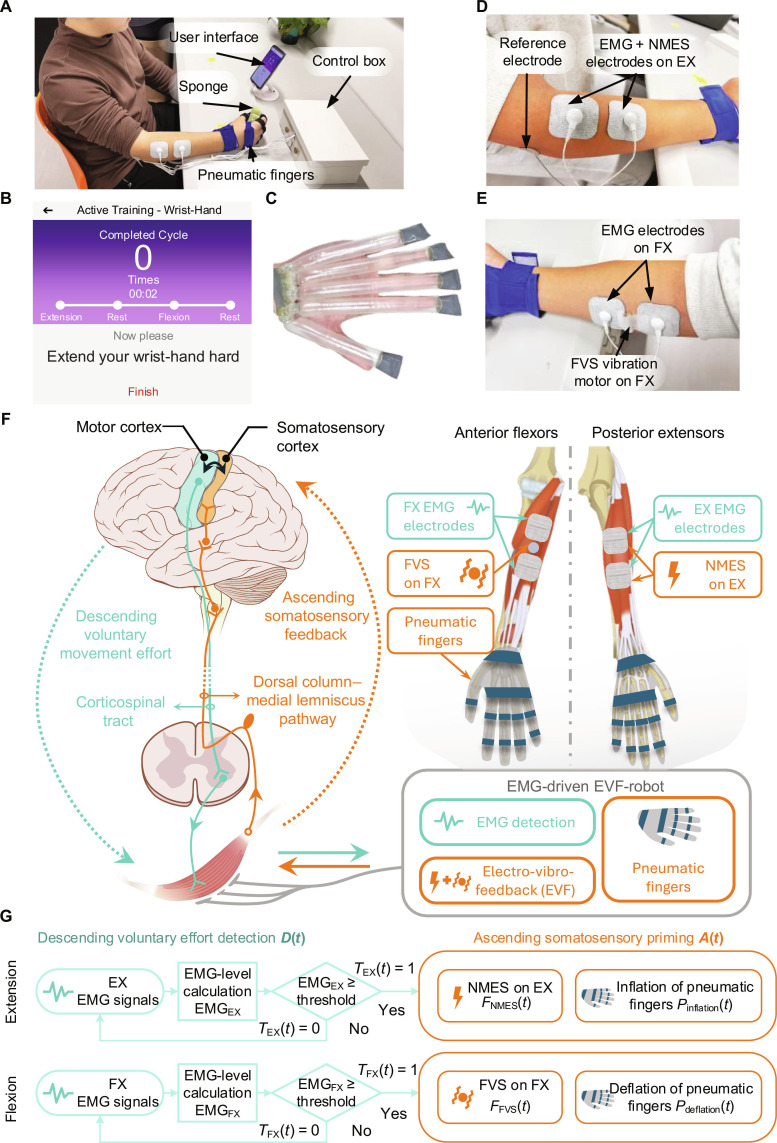
The design of the electromyography (EMG)-driven soft robot with electro-vibro-feedback (EVF-robot). (A) A user wears the EMG-driven EVF-robot. (B) The application on a smartphone serving as a user interface. (C) The pneumatic fingers embedded in an elastic textile glove. The electro-vibro-feedback (EVF) system targeting the (D) extensor digitorum–extensor carpi ulnaris (EX) and (E) flexor digitorum–flexor carpi radialis (FX) muscle unions. (F) Illustration of the sensorimotor integration (SMI) modulating effects on the descending and ascending corticomuscular pathways with a targeted muscle in the SMI control strategy. (G) The SMI control diagram of assistance in phasic wrist/hand (W/H) motions. NMES, neuromuscular electrical stimulation; FVS, focal vibratory stimulation.

Poststroke neuroplasticity for motor recovery with minimized behavioral compensation highly depends on the effective modulation of both descending and ascending corticomuscular pathways; for example, the corticospinal tract (CST) is the main efferent pathway for motor outputs, while the dorsal column-medial lemniscus pathway is the main tract for afferent somatosensory inputs (Fig. 1F) [10–12]. This is because voluntary limb movement requires interactive neural processing of motor control and sensory feedback between the central nervous system and peripheral muscles to achieve movement precision, i.e., sensorimotor integration (SMI) [6,11–13]. However, studies reported that cortical centers governing descending motor commands of the paretic W/H commonly shifted toward the ipsilateral hemisphere in chronic stroke, such as in the control of muscle unions of extensor digitorum–extensor carpi ulnaris (ED–ECU or EX) and flexor digitorum–flexor carpi radialis (FD–FCR or FX) [5,6]. Meanwhile, ascending feedback from EX and FX in the paretic arm exhibited a unilateral cortical response localized to the ipsilateral hemisphere, in contrast to the bilateral activation observed in unimpaired individuals [14]. These cortically derived ipsilateral compensations in descending and ascending pathways were recognized as a primary reason for discoordination among proximal–distal muscles in the paretic UL, as distal muscles received fewer ipsilateral motor projections than proximal muscles [3], leading to reduced specificity of motor control for distal muscles strongly associated with learned disuse in chronic stroke [15].

However, effective methods to restore desired neural circuits for SMI in poststroke rehabilitation are still lacking. Current interventions often overlook the interdependence of descending motor and ascending somatosensory pathways in recruiting target muscles. For example, robots have been widely used to assist physical training by mimicking therapists’ actions for motor rehabilitation [16–18]. While proprioceptive input to the paretic limb can be obtained through continuous joint motion assisted mechanically by robots, these systems predominantly focused on the kinematic accuracy of limb movements, such as trajectory [19] and angular velocity [20,21], rather than achieving desired contraction patterns among targeted muscles [13,17,18]. SMI control remains incomplete in recent robotic control designs, although the descending pathway to a target muscle could be recruited via electromyography (EMG)-driven strategies to trigger robotic assistance as neurofeedback rewards once desired EMG patterns were detected from this muscle [22,23]. However, modulating the ascending pathway from the target muscle for somatosensory feedback has not been well considered in current robots.

Somatosensory priming by stimulation to targeted muscles paired with routine physical limb practice by human therapists yielded better outcomes than the routine alone, because of the additional ascending somatosensory facilitation derived from muscular stimulation [13,24,25]. Clinically, this priming typically involves neuromuscular electrical stimulation (NMES) or focal vibratory stimulation (FVS) applied noninvasively to specific muscles [25]. NMES can promote additional sensory input in addition to its effects of muscle force enhancement and atrophy prevention, such as in distal weak extensors (e.g., EX) during poststroke rehabilitation [10,23,26]. However, NMES directly elicits wide recruitment of diverse sensory receptors in the skin and muscles, which is associated with pain. Moreover, prolonged use of NMES can easily cause muscle fatigue and worsen hypertonia, particularly in spastic flexors (e.g., FX) poststroke [14,27,28]. On the other hand, FVS mainly activates mechanoreceptors within muscles, providing relatively comfortable somatosensory facilitation without exacerbating spasticity [14,29]. Nevertheless, FVS is unable to evoke sustained muscle contraction in a muscle because its mechanical vibration cannot directly depolarize muscle fibers, unlike NMES. Therefore, FVS is commonly applied to spastic muscles in clinical usage [30], rather than to weakened muscles whose voluntary contraction force requires enhancement during poststroke rehabilitation. The integration of NMES with robotic assistance has been designed by our team and others previously to achieve precise limb kinematics and muscle contraction patterns simultaneously in poststroke intervention [23,31]. Nevertheless, the limitations of NMES in recruiting spastic muscles and the associated fatigue constrained effective SMI within desired neuromuscular pathways [13]. It is possible that applying NMES to weak muscles with learned disuse (e.g., EX) while employing FVS for spastic muscles (e.g., FX) in robot-assisted limb practice may optimize SMI, which has not been investigated so far.

In this study, we designed an EMG-driven soft W/H robot with electro-vibro-feedback (EVF-robot) that combines NMES and FVS for targeted SMI modulation in EX and FX muscles after stroke. The EMG-driven EVF-robot employs a novel SMI control to modulate descending and ascending pathways. It detects descending voluntary motor effort (VME) in EX/FX muscles via surface EMG, triggers assistive W/H movements actuated by pneumatic fingers, and concurrently delivers NMES to EX muscles and FVS to FX muscles for targeted somatosensory priming. A single-arm clinical trial investigated the functional efficacy and neuroplastic mechanisms of SMI rehabilitation training using the EMG-driven EVF-robot in participants with chronic stroke. We hypothesized that this approach would enhance ascending somatosensory feedback from target muscles, reinforce descending corticomotor drive, correct maladaptive compensatory neuroplasticity, and improve W/H motor control.

## Materials and Methods

### EMG-driven EVF-robot for targeted somatosensory priming in W/H

The EMG-driven EVF-robot (Fig. 1) is a soft robotic system for targeted SMI modulation in W/H rehabilitation after stroke. It integrates active assistance from 5 pneumatic fingers (Fig. 1C), one-channel NMES (Fig. 1D), and one-channel FVS (Fig. 1E) into one system driven by residual EMG signals detected from the paretic EX and FX muscles in individuals with chronic stroke, based on the NMES-soft robot platform developed by the team previously [23]. Inflation of the pneumatic fingers, together with NMES applied to the EX, assists the paretic W/H to achieve wrist extension coupled with hand-opening motions. Deflation of the pneumatic fingers, paired with FVS applied to the FX, enables the paretic W/H to perform wrist flexion with hand-closing motions.

The real-time SMI control (Fig. 1F and G) was designed to coordinate EMG detection, NMES, FVS, and pneumatic actuation to reinforce targeted corticomuscular pathways during W/H extension and flexion. The assistance of the EMG-driven EVF-robot is defined as follows:

AssistanceEMG−drivenEVF−robot=Dt·At=TEXt·FNMESt+Pinflationt,inW/HextensionTFXt·FFVSt+Pdeflationt,inW/Hflexion (1)

where

Tit=1,ifEMGit≥τi+bi,0,otherwise,i∈EXFX (2)

The parameter $D\left(t\right)$ represents the modulation of the descending pathway (green components in Fig. 1F), in which residual voluntary movement efforts generated by the motor cortex in a stroke participant during W/H extension and flexion is detected by EMG signals ${\mathrm{EMG}}_{i}$ from the paretic EX and FX muscles, respectively, serving as a trigger $T_{i}$ for robotic assistance. EMG signals are full-wave rectified and moving-averaged with a 100-ms window to obtain the EMG activation level during real-time processing [23]. The EMG threshold level $\tau_{i}$ in each motion phase is set to 10% maximal voluntary contraction (MVC) above the EMG baseline $b_{i}$ measured during the resting state to ensure that the signal explicitly indicates voluntary activation of the muscle [32]. This strategy has been implemented in our previous EMG-driven robotic systems to successfully identify VME from an impaired muscle in persons with chronic stroke [26,33,34]. This 10% MVC threshold corresponds to a feasible voluntary contraction level, which effectively activates motor pathways while minimizing fatigue during repeated efforts. The same 10% MVC threshold was applied to both W/H EX and FX to maintain consistent voluntary motor output from the driving muscles, thereby supporting reliable VME detection across all participants. When the voluntary EMG activation level ${\mathrm{EMG}}_{i}\left(t\right)$ on the EX/FX muscles reaches the preset threshold $\tau_{i}$, EX/FX receives ascending somatosensory priming $A\left(t\right)$ from the EVF-robot as neurofeedback rewards for successful voluntary control of the target muscles.

The ascending somatosensory priming $A\left(t\right)$ arises from the coordination of the electro-vibro-feedback (EVF) system, $F\left(t\right)$, and the actuation of the pneumatic fingers, $P\left(t\right)$. The EVF system employs a trigger-mode control to deliver NMES and FVS selectively to the EX and FX muscles, respectively (orange components in Fig. 1F), thereby eliciting joint movements and targeted muscular stimulation. In the W/H extension phase, when robotic assistance is triggered by EMG in EX, i.e., $T_{\mathrm{EX}}\left(t\right)=1$, continuous NMES $F_{\text{NMES}}\left(t\right)$ is applied to EX, while inflated pneumatic fingers $P_{\text{inflation}}\left(t\right)$ mechanically assist fingers to aid in W/H extension throughout the motion phase. In the W/H flexion phase, as soon as robotic assistance is triggered by EMG in FX, i.e., $T_{\mathrm{FX}}\left(t\right)=1$, continuous FVS $F_{\mathrm{FVS}}\left(t\right)$ is delivered to FX, alongside passive deflation of the pneumatic fingers $P_{\text{deflation}}\left(t\right)$ throughout the voluntary W/H flexion. To avoid contamination of EMG signals by NMES or FVS artifacts, EMG detection was performed only during the pre-triggering EMG detection phases, and no EMG was recorded during assistive phases.

For individual user configurations, electrodes (5 cm × 5 cm, UltraStim Snap Electrodes, Axelgaard Manufacturing Co., Ltd., Fallbrook, CA, USA) were attached over the respective EX and FX muscle unions in a bipolar configuration for EMG detection (Fig. 1D and E) [23], with a reference electrode (⌀ 2.5 cm, PALS Platinum Electrodes, Axelgaard) attached to the skin surface of the olecranon. The electrode pair on EX also delivered in situ NMES to the muscle (Fig. 1D). NMES was delivered as bursts of square pulses at 40 Hz (40 pulses per second) with a compliance voltage of 70 V. The intensity of NMES was controlled by adjusting the pulse width, which was continuously variable from 0 to 300 μs, while frequency and amplitude remained constant [23]. For experimental use, the pulse width was titrated individually to achieve maximum functional hand opening without discomfort. FVS was applied to FX using a miniature vibration motor (E0716M, NFP-Motor Co., Ltd, Kowloon, Hong Kong, China) attached to the skin surface in between the EMG electrode pair (Fig. 1E), generating vibrations up to 5.1 G acceleration. The maximum vibration intensity below the pain threshold of each participant was adopted. The pneumatic fingers (Fig. 1C) generated extension torque to facilitate hand opening, with maximum inner pressure limited to 100 kPa according to Nam et al. [23]. A smartphone application (app) (Fig. 1B) served as the user interface to guide phasic motions.

### Experimental protocol of EMG-driven EVF-robot SMI training

After obtaining ethical approval from the Human Subjects Ethics Sub-committee of the Hong Kong Polytechnic University before commencement (approval number: HSEARS20210320003), a single-arm clinical trial involving 15 chronic stroke participants was conducted to investigate the functional efficacy and neurophysiological mechanisms of the SMI UL rehabilitation training program assisted by the EMG-driven EVF-robot. The participant recruitment criteria and configuration of the EMG-driven EVF-robot for participants in the SMI training are detailed in the Supplementary Materials.

**Fig. 2.**
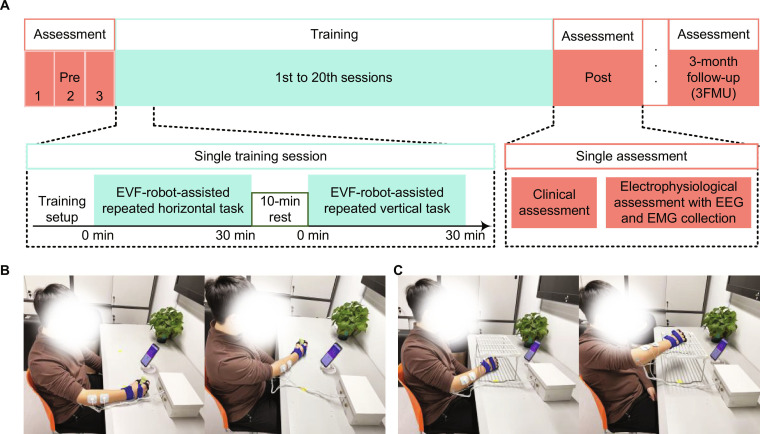
The protocol and setup of the SMI upper limb (UL) rehabilitation training program using the EMG-driven EVF-robot. (A) Protocol of the training program with assessments before (Pre), after (Post), and 3 months after (3MFU) the training sessions. In the Pre-assessment, clinical assessments were conducted 3 times over 2 weeks. Repetitive arm tasks included (B) horizontal tasks and (C) vertical tasks in a training session. EEG, electroencephalogram.

The protocol for the SMI rehabilitation training program using the EMG-driven EVF-robot consisted of 20 sessions (Fig. 2A), each lasting 60 min. Training was scheduled 3 to 5 times per week over 7 consecutive weeks, with a minimum interval of 24 h between consecutive sessions to ensure adequate recovery. A single session consisted of two 30-min functional task blocks (horizontal and vertical tasks) separated by a mandatory 10-min rest interval. The participant was instructed to sit at a table with their shoulders maintained 30 to 40 cm vertically above the table surface (Fig. 2B). A smartphone running the training app was placed on the table in front of the participant. The device was worn on the paretic UL, with EVF electrodes and vibrators attached over target EX/FX muscles after standard skin preparation [35]. System parameters were calibrated for each participant according to the configuration detailed in the Supplementary Materials. Assisted by the EMG-driven EVF-robot, the participant performed horizontal and vertical tasks guided by on-screen instructions as many repetitions as comfortably tolerated at self-selected paces, averaging approximately 45 trials per block, as detailed in our previous study [36]. In brief, the horizontal task simulated table-top object transfer (e.g., passing a cup), while the vertical task mimicked shelf-reaching motions to train functional workspace utilization in daily life. For the horizontal task (Fig. 2B), the participant was instructed to grasp a sponge (5 cm thick, 30-g mass) near the affected side, move it 50 cm horizontally to the unaffected side, release it, then grasp and return it to the starting position, and release it again. For the vertical task (Fig. 2C), the participant was instructed to lift the sponge from the table to a shelf at a 18-cm height, release it, then bring it back down, and place it on the table before releasing it again. Although the horizontal and vertical training tasks incorporated various wrist and forearm movements, the robotic assistance was primarily targeted at W/H grasping and releasing, which are essential for daily functional tasks. The assistance was muscle specific, phase-locked, and individually adjusted in intensity/frequency, synchronized precisely with EMG signals to ensure support only during the appropriate phase of movement. In addition, participants were monitored for adverse events and fatigue during the training. Noncompliant participants would be excluded from the study.

### Assessment of sensorimotor functions and corticomuscular neuroplasticity

To investigate the functional efficacy and neuroplastic mechanisms of SMI rehabilitation training using the EMG-driven EVF-robot in chronic stroke participants, clinical and electrophysiological assessments were conducted at 3 time points: before training (Pre), 1 d after training (Post), and 3-month follow-up (3MFU) (Fig. 2A).

#### Clinical assessments of UL sensorimotor functions

The adopted clinical assessments included (a) motor functional assessment in voluntary limb movements by the Fugl-Meyer Assessment (FMA), with a total score of 66 for the UL portion (FMA-upper extremity [FMA-UE]) [37]; (b) assessment of the UL voluntary functions focusing on the functional tasks by the Action Research Arm Test (ARAT) [38]; (c) sensation assessment on the affected arm using the Semmes–Weinstein monofilament test [39], applied on the skin surface over the EX and FX muscles and 6 sites on the ventral and dorsal sides of the hand, following an established protocol [40]; and (d) muscle spasticity assessment at the elbow, wrist, and finger joints measured by Modified Ashworth Scale (MAS) [41].

During the Pre-assessment, motor clinical evaluations (FMA, ARAT, and MAS) were conducted 3 times over a 2-week period to ensure baseline stability, and the averaged values were used for statistical analysis. In contrast, the monofilament sensation test was performed once before training, as its primary purpose was to capture changes in tactile sensitivity between the pre- and post-states. Post and 3MFU assessments included the same set of clinical measures. All clinical assessments were conducted by an assessor blinded to the training protocol.

#### Assessments of intermuscular coordination and corticomuscular neuroplasticity

EMG and electroencephalogram (EEG) signals were collected at the 3 assessment time points (Pre, Post, and 3MFU) to analyze the intermuscular coherence (IMC), corticomuscular coherence (CMC), and pathway-specific directed CMC (dCMC) of UL muscles. IMC reflects the common central drive to the motor units of synergistic muscles (i.e., muscular co-activation) [42]. CMC and dCMC measure the coherence between EEG and EMG signals, which reveals time-dependent functional connections in neuromuscular pathways between the cortex and target muscles during specific motion tasks [5,42,43].

For CMC and dCMC assessment, a 64-channel EEG cap was mounted on the scalp of the participant according to the standard 10–10 system [44]. EEG signals from 21 electrodes (CZ, CPZ, FCZ, C1 to C6, CP1 to CP6, and FC1 to FC6) covering the sensorimotor cortex were selected for analysis. EMG electrodes were attached to the target UL muscles, i.e., EX, FX, biceps brachii (BIC), and triceps brachii (TRI), after standard skin preparation [35]. The participant performed 5 trials of isometric W/H extension and flexion at 20% MVC for EX and FX, respectively, following a protocol previously applied to stroke survivors [3,5]. No NMES, FVS, or robotic pneumatic assistance was applied during this evaluation session, ensuring that the observed responses reflected training-induced neuroplasticity rather than immediate stimulation effects. EEG and EMG signals were recorded simultaneously during each trial. Before movement onset, 10 s of resting-state EEG signals were recorded for off-line baseline correction. After movement onset, participants maintained 20% MVC for EX or FX for 35 s. For IMC assessment, the participant performed horizontal and vertical tasks (identical to the training session) 3 times without the device (Fig. 2B and C). EMG signals from EX, FX, BIC, and TRI were recorded during evaluation. All EEG or EMG signals were recorded at a sampling rate of 1,200 Hz using the same amplifiers (g.USBamp, g.tec, USA).

**Fig. 3.**
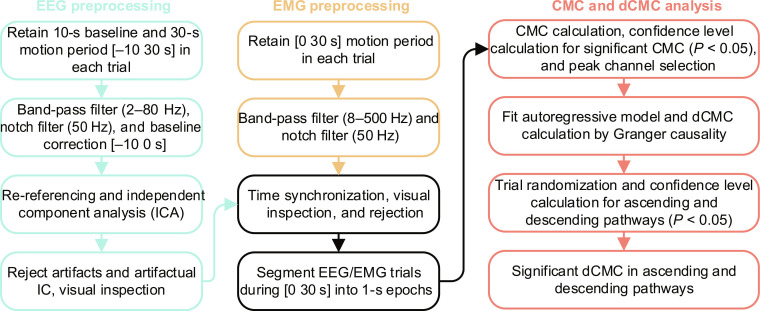
Signal processing flowchart for corticomuscular coherence (CMC) and directed CMC (dCMC) analysis.

The procedures of off-line EEG/EMG preprocessing and further analysis are shown in Fig. 3. For EEG preprocessing, the raw EEG data were retained [−10 30 s], where 0 s denotes the motion onset. Next, EEG signals were band-pass filtered at 2 to 80 Hz, notch-filtered at 50 Hz, corrected using the baseline period [−10 0 s], re-referenced to the average of all channels, and analyzed by independent component analysis to identify components with artifacts [45,46]. Visual inspection of the EEG signals was performed to remove trials with residual artifacts. For EMG preprocessing, the raw EMG data during the motion period [0 30 s] were band-pass filtered at 8 to 500 Hz and notch-filtered at 50 Hz. After preprocessing, synchronized EEG and EMG data for CMC analysis were segmented into 1-s epochs during the motion period [0 30 s]. These preprocessing and analysis steps followed standard procedures in the FieldTrip [47] and EEGLAB [48] toolboxes, implemented with the latest updates using MATLAB 2024a (MathWorks, Natick, MA, USA).

IMC in the alpha (8 to 13 Hz), beta (13 to 30 Hz), and gamma (30 to 80 Hz) frequency bands was analyzed among UL target muscles (EX, FX, BIC, and TRI) [49]. CMC in the beta band was analyzed to identify significant coherence between the 21 channels over the sensorimotor cortex and UL target muscles in each participant. The IMC or CMC of each EMG–EMG or EEG–EMG pair was calculated using

$$\text{Coherence}\left(\sigma \right)=\frac{{\left|f_{\mathrm{xy}}\left(\sigma \right)\right|}^{2}}{f_{\mathrm{xx}}\left(\sigma \right)\cdot f_{\mathrm{yy}}\left(\sigma \right)}$$ (3)

where *f_xx_*(*σ*) and *f_yy_*(*σ*) represent the auto-spectrum of each signal, respectively, and *f_xy_*(*σ*) represents the cross-spectrum of the 2 paired signals [42,50]. The CMC amplitude exceeding the 95% confidence level ${\mathrm{CL}}_{95\%}=1-0.05^{\frac{1}{n-1}}$ was considered significant, where *n* is the number of epochs used for the CMC calculation [5]. To investigate cortical reorganization after training, the laterality index (LI) of CMC topography was calculated using

$$\mathrm{LI}=\frac{\max\left({\mathrm{CMC}}_{\text{contralateral}}\right)-\max\left({\mathrm{CMC}}_{\text{ipsilateral}}\right)}{\max\left({\mathrm{CMC}}_{\text{contralateral}}\right)+\max\left({\mathrm{CMC}}_{\text{ipsilateral}}\right)}$$ (4)

where $\max\left({\mathrm{CMC}}_{h}\right)$ denotes the highest significant CMC amplitude (i.e., the peak CMC) among EEG channels over the hemisphere *h* [51]. A positive LI indicates contralateral dominance in CMC topography, while a negative LI reflects ipsilateral dominance. The EEG channel exhibiting the peak CMC amplitude was identified as the channel of interest for subsequent dCMC analysis [3].

dCMC was calculated using Granger causality, a frequency-domain directional estimator based on autoregressive (AR) modeling. A bivariate AR model with order *k* was fitted at time *t* for each EEG–EMG pair using

$$\sum_{\tau =0}^{k}A_{\tau }X_{t-\tau }=E_{t}$$ (5)

where ***A****_τ_* is a 2 × 2 coefficient matrix, ***X****_t_* = (EEG*_t_*, EMG*_t_*)^T^ is the 2 × 1 signal matrix, ***E****_t_* is the residual error with covariance matrix ***C***, and *τ* is the time delay [52]. An AR model with order *k* = 60 was selected and validated to ensure sufficient spectral resolution, consistency, stability, and whiteness of residuals [53], following prior implementations [3]. The frequency-domain transfer function $H\left(f\right)$ was then computed as

$$H\left(f\right)={\left(\sum_{\tau =0}^{k}A_{\tau }e^{-2\pi \mathrm{i\tau f}}\right)}^{-1}$$ (6)

where *i* is the imaginary unit [52]. Finally, the dCMC from signal 1 to signal 2 in ***X****_t_* (EEG → EMG) at frequency *f* was calculated using frequency-domain Granger causality:

$${\text{dCMC}}_{1\to 2}\left(f\right)=-\ln\left(1-\frac{\left(C_{11}-\frac{C_{12}^{2}}{C_{22}}\right){\left|H_{21}\left(f\right)\right|}^{2}}{S_{22}\left(f\right)}\right)$$ (7)

where *C*_11_, *C*_12_, and *C*_22_ are elements of ***C***; *H*_21_(*f*) quantifies the connection from signal 1 to signal 2 (EEG → EMG); and *S*_22_(*f*) is the power spectrum of signal 2 (EMG) at frequency *f* [52,54]. Similarly, the reverse dCMC from signal 2 to signal 1 (EMG → EEG) was computed by swapping subscripts in Eq. (7). Significance thresholds for dCMC were determined via nonparametric Monte Carlo simulations. Surrogate datasets were generated by randomly shuffling trials of the original EEG and EMG signals across 1,000 iterations. The 95th percentile of the surrogate dCMC distribution defined the significant threshold (*P* < 0.05), minimizing false positives [55,56]. Nonsignificant dCMC values were set to zero for subsequent statistical analysis.

### Statistical analysis

The Shapiro–Wilk normality test was used to evaluate the normality of clinical scores and IMC, CMC LI, and dCMC parameters at a significant level of 0.05. Only MAS and dCMC amplitude violated normality (*P* < 0.05). Other parameters followed a normal distribution (*P* > 0.05). Thus, for MAS and dCMC amplitude, we applied the nonparametric Friedman test with false discovery rate (FDR) correction to assess changes across the Pre, Post, and 3MFU time points. For other normally distributed parameters, one-way analysis of variance (ANOVA) with repeated measures and Tukey’s post hoc test for multiple comparison corrections were adopted. In addition, baseline Pre-assessment scores showed no significant difference (*P* > 0.05), confirming a stable rehabilitative baseline. Baseline scores were thus averaged into a single Pre-assessment value for subsequent analysis. Furthermore, stratified comparisons were performed to account for population heterogeneity. Participants were grouped by stroke chronicity (≤5 years vs. ≥5 years) and by baseline impairment level according to FMA-UE scores. Nonparametric tests (Mann–Whitney *U*) were used to examine FMA-UE differences in training effects across strata. Statistical analyses in this study were performed using GraphPad Prism 9.5. The significance level was set at *P* < 0.05, with *P* < 0.01 also reported.

## Results

All recruited participants (*N* = 15) completed the SMI rehabilitation training program, assisted by the EMG-driven EVF-robot, as well as all assessments. The demographic information of the participants is shown in Table 1, including stratification by baseline impairment level based on FMA-UE scores and stroke chronicity (≤5 years vs. ≥5 years).

**Table 1.** Demographic information of the participants

Characteristics	Values
Stroke type (ischemic/hemorrhagic)	3/12
Affected side (right/left)	5/10
Gender (male/female)	7/8
Age in years (mean ± SD)	52.93 ± 11.25
Time since stroke in years (mean ± SD)	7.42 ± 6.92
<5 years (*n* = 9)	2.44 ± 1.13
≥5 years (*n* = 6)	13.67 ± 6.25
Initial impairment level by FMA-UE (mean ± SD)	33.09 ± 9.43
15 < FMA-UE ≤ 30 (*n* = 7)	23.71 ± 4.24
30 < FMA-UE < 45 (*n* = 8)	41.29 ± 3.25

FMA-UE, Fugl-Meyer Assessment-upper extremity

### Changes in UL sensorimotor functions after the SMI rehabilitation training

**Fig. 4.**
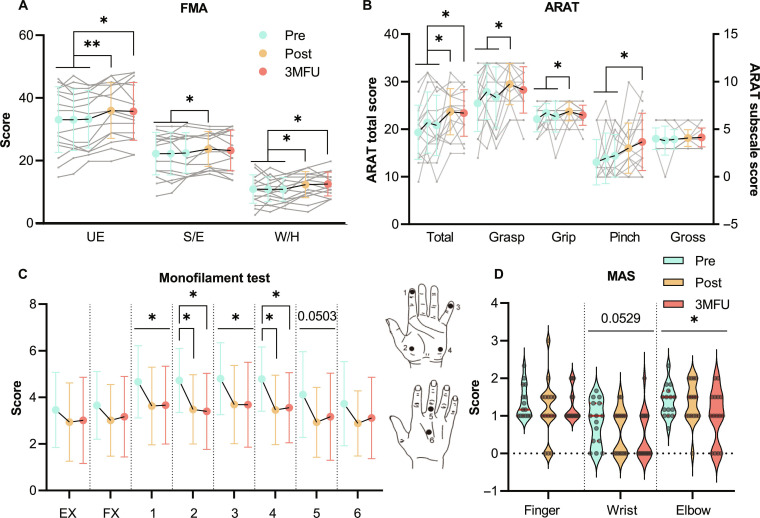
Clinical scores across evaluation time points. (A) Fugl-Meyer Assessment (FMA) scores. (B) Action Research Arm Test (ARAT) total and subscale scores. (C) Monofilament test scores. (D) Modified Ashworth Scale (MAS) scores. Pre-assessments were conducted 3 times and averaged for statistical analysis. Results are presented as mean ± SD in (A), (B), and (C) and violin plots in (D). Individual participant values are shown as data points connected by lines over time in (A) and (B). Significant differences are denoted by “*” for *P* < 0.05 and “**” for *P* < 0.01. UE, upper extremity; S/E, shoulder/elbow.

Significant increases were found after the training in FMA-UE total, FMA-S/E, and FMA-W/H scores and ARAT total, grasp, and grip scores (Fig. 4A and B and Table S2, corrected *P* = 0.0023, 0.0464, 0.0320, 0.0485, 0.0465, and 0.0292, one-way ANOVA with Tukey’s post hoc test). Significant increases were preserved at 3MFU in FMA-UE, FMA-W/H, and ARAT total and pinch scores (corrected *P* = 0.0367, 0.0360, 0.0133, and 0.0483, one-way ANOVA with Tukey’s post hoc test).

In monofilament assessment (Fig. 4C and Table S3), sites 1 to 4 are on the ventral side of the hand, and sites 5 and 6 are on the dorsal side; sites 1 and 2, 3 and 4, and 5 and 6 are innervated by the median, ulnar, and radial nerves, respectively [40]. Significant decreases in monofilament scores at sites 2 and 4 were observed after training and persisted at 3MFU (Post vs. Pre: corrected *P* = 0.0190 and 0.0182; 3MFU vs. Pre: corrected *P* = 0.0059 and 0.0092, one-way ANOVA with Tukey’s post hoc test). Significant decreases over time were observed at sites 1 and 3 (*P* = 0.0432 and 0.0263, one-way ANOVA) but not with Tukey’s post hoc test. Average monofilament scores at site 5 decreased over time (*P* = 0.0503, one-way ANOVA), showing marginal significance.

Significant decreases in MAS scores were found over time in the elbow joint (Fig. 4D and Table S4, *P* = 0.0289, Friedman test) but not with the post hoc test after FDR correction. Average MAS scores in the wrist joint decreased over time (*P* = 0.0548, Friedman test), showing marginal significance. Other parameters not marked in Fig. 4 exhibited no significant changes over time.

Stratified analyses revealed no statistically significant differences in FMA-UE improvement between participants with shorter versus longer chronicity, or across different baseline severity levels (Table S5, *P* > 0.05, Mann–Whitney *U* test). These findings suggest that the observed training effects were consistent across subgroups.

### Changes in intermuscular coordination after the SMI rehabilitation training

**Fig. 5.**
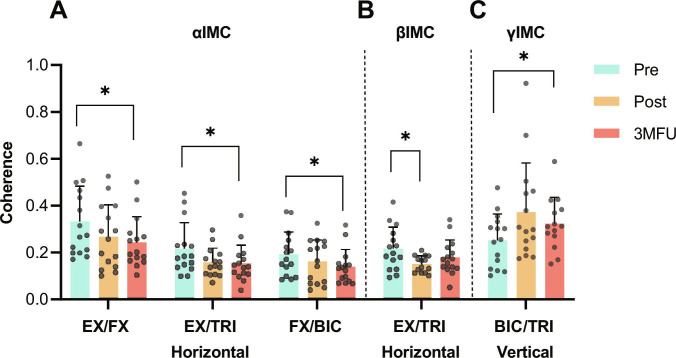
Significant changes in intermuscular coherence (IMC) between UL muscle pairs across evaluation time points in alpha (A), beta (B), and gamma bands (C) (mean ± SD with all data points). Significant changes are denoted by “*” for *P* < 0.05. αIMC, alpha IMC; βIMC, beta IMC; γIMC, gamma IMC; TRI, triceps brachii; BIC, biceps brachii.

Significant decreases in alpha IMC (αIMC, Fig. 5A) were detected in the EX/FX, EX/TRI, and FX/BIC muscle pairs at 3MFU during the horizontal task (corrected *P* = 0.0331, 0.0212, and 0.0334, one-way ANOVA with Tukey’s post hoc test). A significant decrease in beta IMC (βIMC, Fig. 5B) was detected in the EX/TRI muscle pair after training during the horizontal task (corrected *P* = 0.0406, one-way ANOVA with Tukey’s post hoc test). A significant increase in gamma IMC (γIMC, Fig. 5C) was detected in the BIC/TRI muscle pair at 3MFU during the vertical task (corrected *P* = 0.0418, one-way ANOVA with Tukey’s post hoc test). No significant changes were observed in IMC for other muscle pairs.

### Changes in corticomuscular neuroplasticity after the SMI rehabilitation training

**Fig. 6.**
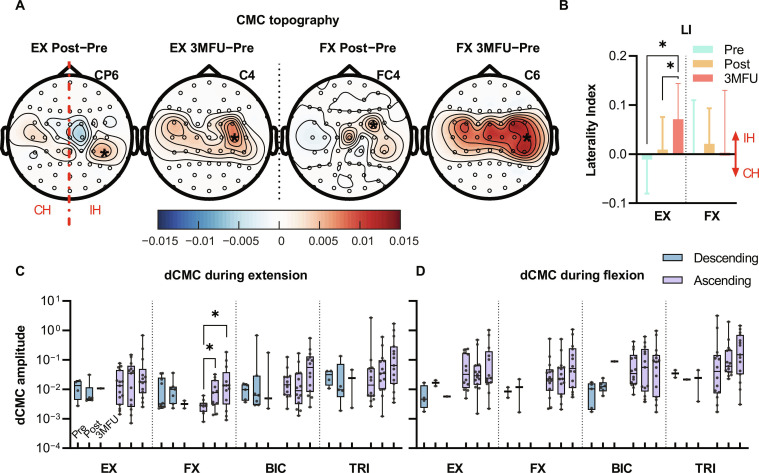
Changes in corticomuscular assessments over time. (A) Grand-averaged topography changes of significant CMC between the sensorimotor cortex and EX/FX muscles during W/H extension/flexion for all participants (*N* = 15) at Post and 3MFU assessments compared with those at the Pre time point. Peak channels are indicated by “*” with corresponding labels in the top right corner of each topography. Note: IH, ipsilesional hemisphere; CH, contralesional hemisphere. (B) Laterality index (LI) changes of significant CMC topography with agonist muscles (mean ± SD). (C and D) Significant dCMC between the peak CMC channel and 4 UL muscles (box-and-whisker plots with significant data points) during W/H extension (C) and flexion (D). Significant changes are denoted by “*” for *P* < 0.05.

In the significant CMC with EX during W/H extension (Fig. 6A, topographies 1 and 2), CMC increased bilaterally with a peak at the CP6 channel in the Post–Pre comparison and at the C4 channel in the 3MFU–Pre comparison. In the significant CMC with FX during W/H flexion (Fig. 6A, topographies 3 and 4), CMC increased in the contralateral/ipsilesional hemisphere, peaking at the FC4 channel in the Post–Pre comparison, and increased bilaterally with a peak at the C6 channel in the 3MFU–Pre comparison. The LI of significant CMC with EX and FX exhibited a shift trend toward contralateral/ipsilesional hemispheric dominance in Post assessments (Fig. 6B). Significant LI shifts toward the contralateral hemisphere were observed in CMC with EX at the 3MFU stage compared to the Pre and Post time points (corrected *P* = 0.0107 and 0.0431, one-way ANOVA with Tukey’s post hoc test).

Only significant dCMC amplitudes of each participant during W/H extension and flexion are shown as data points in Fig. 6C and D, respectively. Improvements in significant dCMC occurred mainly in the ascending pathway, rather than the descending pathway, across all conditions and time points. Significant increases in ascending dCMC with FX muscles during W/H extension were observed after training and persisted at 3MFU (Fig. 6C, corrected *P* = 0.0149 and 0.0004, Friedman test with FDR correction). No significant changes in dCMC were observed in other muscles of the descending or ascending pathways during extension or flexion.

## Discussion

In this study, a novel EMG-driven EVF-robot for SMI W/H rehabilitation after stroke was designed. NMES was applied to EX and FVS to FX, reflecting their distinct poststroke impairments. Extensors are typically weak and underrecruited after stroke, making NMES an appropriate intervention to strengthen muscle force and promote functional hand opening, as motor-level NMES can evoke muscle contraction by depolarizing muscle fibers electrically [14]. In contrast, flexors often preserve better residual voluntary motor control than extensors. However, they usually develop spasticity with involuntary contractures in the chronic phase of stroke, and the use of NMES may exacerbate hypertonia and induce muscle fatigue [28]. The FVS applied to the target muscle in this study mainly activates mechanoreceptors for sensory input without depolarizing muscle fibers [30,57], which could enhance the ascending pathway for the targeted FX without exacerbating spasticity. Feasibility was validated in the clinical trial, where all 15 participants completed the SMI rehabilitation training without dropout, adverse events, or fatigue. The EMG spectral analysis during training confirmed that the mean power frequency of the target muscles remained stable across each EMG trigger event, with no reduction exceeding 10% across repetitions. This suggests the absence of fatigue-related spectral shifts throughout the intervention [58]. Building on this feasibility, results demonstrated improved W/H motor control via enhancing somatosensory feedback from target muscles, promoting correction of compensatory neuroplasticity with effects persisting for 3 months.

### Rehabilitative efficacy of the SMI training

The EMG-driven EVF-robot produced durable motor and somatosensory gains in the UL, particularly in distal W/H joints. While FMA scores showed broad UL motor improvement posttraining (Fig. 4A), W/H joint control surpassed S/E outcomes at 3MFU. ARAT scores highlighted significant W/H fine motor improvements during functional tasks such as grasping and pinching (Fig. 4B). These results contrast with prior robotic UL rehabilitation strategies, which showed poorer long-term distal joint recovery [22], likely due to inadequate modulation of somatosensory pathways [4]. Although NMES-robots improved motor function clinically, they failed to incorporate SMI designs targeting somatosensory pathways or to validate their efficacy on the somatosensory system [23,36,59]. Previous FVS studies attributed joint range-of-motion improvements to spasticity reduction after interventions, mediated through vibration-induced Ia spindle activation [30,57]. In this work, W/H spasticity reduction was only marginally significant (Fig. 4D), compared to the significant decrease in the upper arm. This implies that the release of W/H spasticity was not the key factor related to the voluntary motor improvements in distal joints. Instead, cutaneous sensitivity significantly improved in median/ulnar-nerve-innervated regions (Fig. 4C, sites 1 to 4), adjacent to FX muscles. Furthermore, FX muscles, as key agonists in ARAT tasks, contributed to fine motor control gains (Fig. 4B). This likely suggests that FVS enhanced proprioceptive feedback in FX muscles, driving neuroplasticity that linked somatosensory facilitation to lasting functional motor gains.

From a clinical perspective, FMA and ARAT scores demonstrated statistically significant improvements over time, but the mean changes did not exceed the minimal clinically important difference (MCID) thresholds, which are context specific and primarily validated in subacute stroke rehabilitation [60,61]. Individuals with chronic stroke typically achieve smaller functional gains after rehabilitation, as clinical practice emphasizes maintaining residual motor functions rather than restoring substantial improvements seen in earlier stages [62]. In our cohort, 5 out of 15 participants with chronic stroke (33.3%) achieved the MCID threshold for FMA-UE, defined as an improvement exceeding 4.25 points adopted in chronic stroke [63], and 3 out of 15 participants (20.0%) achieved the MCID for ARAT, with a threshold set at 5.7 points [64]. It was also noted that the training dosage in our study (20 sessions, 3 to 5 sessions per week, 60 min per session) was relatively modest compared to the protocols described by Page et al. [63] and van der Lee et al. [64]. For instance, patients in Page et al. [63] underwent 26 d of repetitive task-specific training over 6 weeks, receiving 2.5 h of training per weekday, combined with implanted cortical stimulation via the Northstar Stroke Recovery System. Similarly, in the forced-use trial reported by van der Lee et al. [64], participants engaged in 6 h of daily training, 5 d per week, for 2 consecutive weeks. The substantially greater training intensity, particularly when augmented with epidural electrical stimulation, may partially account for the larger MCID observed in those studies. Given the exploratory nature of this study, the optimal training duration may have been underestimated. Moreover, the motor gains in this study were primarily observed in distal W/H function, as evidenced by significant improvements in specific subdomains such as the FMA-W/H and the pinch and grasp subscores of ARAT, indicating targeted therapeutic benefits despite modest total score changes. In our repeated-measures ANOVA, the effect sizes for both FMA-UE and ARAT exceeded 0.3 (Table S2), surpassing the conventional threshold for a large effect size (partial *η*^2^ = 0.14) [65], which supports the robustness of the observed intervention effect. These results suggest that even with a moderate training intensity, targeted gains in distal function can be achieved, which may hold clinical value for maintaining independence in daily activities.

Although the cohort included strokes with broad chronicity and varying baseline severity, stratified analyses indicated that the training effects were independent of stroke duration and initial impairment. Neither earlier onset nor higher baseline function was associated with greater recovery in this cohort (Table S5). This suggests that the intervention may exert consistent benefits across heterogeneous patient populations, although larger studies are warranted to confirm these findings.

### Reduced UL intermuscular compensation

The EMG-driven EVF-robot was designed to engage in descending voluntary effort and ascending sensorimotor feedback from targeted forearm extensors and flexors when assisting meaningful UL tasks, e.g., the horizontal and vertical tasks of the study, thereby progressively minimizing proximal compensation. Chronic stroke patients commonly exhibit proximal compensation from S/E regions even during isolated isometric contractions of forearm muscles without overt joint motion [3,5], where independent volitional control of W/H muscles becomes replaced by compensatory proximal muscle activation patterns, leading to “learned disuse” in poststroke chronic stage [2,4]. The participants demonstrated reduced intermuscular compensation and improved muscle synergy among the paretic UL muscles after the SMI training. The participants displayed enhanced coordination between the agonist–antagonist (EX/FX) and proximal–distal (EX/TRI and FX/BIC) muscle pairs, which may relate to increased spinal inhibition associated with the reduced αIMC (Fig. 5A) [42], mainly introduced by FVS as reported by Rocchi et al. [66]. Additionally, reduced βIMC (Fig. 5B) indicated less co-activation and greater selectivity in proximal–distal pairs (EX/TRI) [42], while increased γIMC (Fig. 5C) reflected lower kinematic error and energy cost in proximal muscles (BIC/TRI) [67]. These IMC changes could be interpreted as refinements of motor control. For example, the synergy of the EX/TRI muscle pair does not represent pathological co-activation but a functional pattern that contributes to inhibiting flexor dominance. Thus, the observed decrease in αIMC may indicate reduced reliance on strong proximal–distal coupling, allowing more selective recruitment of distal extensors [42,68]. Similarly, the increase in γIMC, observed in the BIC/TRI pair, is consistent with dynamically regulated contractions [42,68]. Although this may reflect compensatory proximal involvement, it still supports movement stability and enables patients to accomplish functional tasks. Consistent with this, lower IMC values indicated diminished recruitment of shoulder stabilizers during wrist-focused tasks, suggesting enhanced neuromuscular selectivity and more refined motor control. Although previous NMES-robot studies achieved reductions in poststroke co-activation similar to those observed in this work, they only superficially reported decreases in muscular spasticity and changes in muscular co-contraction patterns [23,36].

By coupling descending voluntary effort with ascending sensorimotor feedback from forearm extensors and flexors, the EMG-driven EVF-robot progressively minimized proximal compensation and promoted more physiologically appropriate activation, via 2 pathways: (a) enhancing spinal inhibition to regulate descending motor commands and (b) correcting compensatory strategies rooted in inefficient neuromuscular control. These improvements in intermuscular compensation further suggested enhanced W/H motor control (via FMA-W/H and ARAT scores; Fig. 4A and B). By translating gains to daily tasks and mitigating W/H learned disuse, this approach fosters sustained neuroplastic recovery.

### Enhanced corticomuscular efficiency with W/H muscles

The SMI training assisted by the EMG-driven EVF-robot promoted contralateral neuroplasticity at the cortical level by reinforcing ascending somatosensory feedback through targeted muscular stimulation in the peripheral, as evidenced by the CMC and dCMC results, ultimately improving motor control of paretic W/H after rehabilitation.

The EMG-driven EVF-robot reduced reliance on ipsilateral compensation in agonist muscle control, restoring proximal–distal coordination. After NMES or FVS stimulated EX/FX muscles during W/H extension or flexion, CMC peaks (Fig. 6A) and LI (Fig. 6B) shifted toward the contralateral hemisphere. Since CMC identifies cortical sources involved in voluntary motor control [5,50], these shifts indicate restored ipsilesional control and enhanced recruitment of the ipsilesional CST disrupted after stroke. This aligns with previous studies on stroke interventions evaluated by functional MRI [69] or CMC [51], contrasting with stroke-induced ipsilateral shifts caused by synaptic pruning and new synaptogenesis [5,6]. By correcting ipsilateral compensations in sensorimotor pathways, this intervention improved coordination between proximal and distal muscles in the paretic UL, as shown in the IMC results (Fig. 5A and B), since distal muscles depend less on ipsilateral projections than proximal muscles [3]. This mechanism may further explain gains in distal joint function [15], as evidenced by higher FMA-W/H and ARAT scores (Fig. 4A and B).

Targeted somatosensory priming by FVS on peripheral FX muscles strengthened ascending somatosensory pathways from distal flexors to the contralateral sensorimotor cortex, promoting contralateral cortical control during SMI training. Crucially, participants exhibited increased ascending dCMC during W/H extension (Fig. 6C) between the FX antagonist muscle and CMC peaks in contralateral hemisphere (Fig. 6A, topographies 1 and 2), demonstrating enhanced somatosensory feedback from muscles to the contralateral sensorimotor cortex [3,70]. These results suggest that repetitive FVS-mediated somatosensory priming targeting FX during SMI training activated ascending pathways to the contralateral sensorimotor cortex, driving improved contralateral motor control in W/H movements. Previous robotic systems struggled to restore paretic UL distal muscles [22], likely because they failed to modulate impaired ascending pathways from targeted peripheral distal muscles to the contralateral sensorimotor cortex, which perpetuated maladaptive ipsilateral compensation [4]. In contrast, the EVF-robot’s FVS overcame this limitation by selectively enhancing FX-related ascending pathways, facilitating a switch from ipsilateral to contralateral control. These findings align with the neuroplasticity benefits of somatosensory priming, which induces reorganization in the sensorimotor cortex to improve rehabilitation efficacy [24,25].

In addition, targeted somatosensory priming with FVS applied to FX muscles improved W/H sensorimotor function during SMI training. Improved monofilament scores (Fig. 4C, sites 1 to 4) and ARAT subscale scores (Fig. 4B) confirmed that W/H sensorimotor improvement resulted from FVS-driven ascending facilitation in FX muscles. Previous studies demonstrated that vibration-based priming of antagonist muscles enhanced W/H extension, although the underlying mechanism remained unclear [71]. We found that FVS priming boosted somatosensory signals from antagonist muscles to the contralateral sensorimotor cortex during extension, likely because FVS triggers transient involuntary attention toward FX muscles and activates the ipsilesional sensorimotor cortex [14]. By repetitively pairing FVS neuromodulation with EMG-triggered robotic movement assistance, somatosensory feedback from FX was strengthened, functionally enhancing the interaction with motor activities (i.e., SMI) and thereby improving W/H sensorimotor function [12,24].

The observed task-dependent effect may be attributed to the asymmetry between flexion and extension functions in individuals with chronic stroke. Flexion function is typically better preserved, whereas extension is more severely impaired. During attempted extension, stroke survivors frequently exhibit pronounced co-contraction of FX, which interferes with the selective activation of EX [7]. By applying FVS to FX, ascending dCMC improved, enhancing cortical control and reducing inappropriate flexor activation. This reduction in maladaptive co-contraction enabled more selective recruitment of EX, resulting in the normalization of the LI and improved voluntary motor execution during extension tasks. In contrast, flexion movements did not demonstrate similar improvements, likely because the flexion capacity was already relatively intact and less reliant on the suppression of aberrant co-contraction. These findings align with previous reports indicating that FVS enhances sensory afferent feedback and promotes neuroplasticity in poststroke rehabilitation [72,73].

However, weak descending motor commands require more effective neuromodulation of descending pathways in SMI rehabilitation after stroke. We found that poststroke dCMC patterns exhibited sparser and weaker descending connections but more numerous and stronger ascending connections after training (Fig. 6C and D). This mismatch suggests that sensory and motor circuits recover at different rates. It supports prior evidence of more impaired descending control over distal UL muscles than ascending feedback during W/H extension poststroke [3], likely due to CST denervation and weakened monosynaptic connections. Our robot-assisted rehabilitation uses EMG-driven control to couple residual motor commands with robotic assistance, maximizing voluntary effort and enhancing corticospinal drive. This control strategy is designed to promote descending pathway activation and improve motor output by engaging spared corticospinal fibers and supporting task-specific cortical reorganization [6,23]. Crucially, enhancing descending control during cortical reorganization remains challenging: Newly reinnervated CSTs in cortical reorganization that shift toward contralateral dominance (Fig. 6A and B) may lack the strength to simultaneously establish descending monosynaptic connections during intervention. To further strengthen descending pathways, future iterations could integrate more effective neuromodulation approaches. In addition to paired central–peripheral stimulation, noninvasive brain stimulation techniques such as transcranial direct current stimulation and transcranial magnetic stimulation may be utilized to increase cortical excitability and boost descending motor signals [13]. Combining these neuromodulatory interventions with VME-driven robotic training may establish a synergistic framework for addressing the imbalance between sensory and motor recovery.

### FVS-enhanced proprioceptive feedback

Tactile sensation and proprioception are distinct sensory modalities, yet accumulating evidence indicates that cutaneous afferents can modulate muscle spindle activity and thereby contribute to proprioceptive integration [74]. In stroke survivors, muscular discoordination is common, as patients frequently recruit nontarget or inappropriate muscles during voluntary movements [3,8,9]. These maladaptive motor patterns highlight the importance of reinforcing precise proprioceptive feedback from target muscles. In this study, NMES or FVS was applied to the appropriate target muscles during voluntary W/H movements, thereby enhancing afferent input from the activated muscles. This augmented sensory feedback strengthened the cortical representations of their anatomical locations and improved motor selectivity [11,27,73]. Therefore, although tactile sensitivity and proprioception are not equivalent, enhanced cutaneous feedback within the median and ulnar nerve territories may augment proprioceptive signaling from associated muscles, supporting neuroplastic changes that reduce maladaptive muscle discoordination.

Furthermore, repeated NMES and FVS applied in somatosensory priming induced neuroplastic changes mirroring their acute neuromodulatory effects. Specifically, CMC topography analysis revealed divergent training effects on EX and FX muscles (Fig. 6A), due to inherent differences between NMES and FVS. In NMES-primed EX muscles, CMC increased bilaterally in the sensorimotor cortex, whereas FVS-primed FX muscles exhibited enhancement only in the contralateral cortex (Fig. 6A, topographies 1 and 3). Additionally, NMES-primed EX muscles lacked ascending facilitation effects, but FVS-primed FX muscles demonstrated significantly enhanced ascending facilitation effects (Fig. 6C and D). The observed increase in afferent signaling from the FD during wrist extension demonstrated training-induced neuroplasticity after the device-assisted intervention. Mechanistically, FVS directly activates flexor muscle spindles, specifically Ia afferents, thereby enhancing activity in ascending sensory pathways and strengthening cortical afferent connectivity [14]. In contrast, NMES primarily elicits motor output from the extensors and exerts minimal influence on flexor sensory pathways [43]. These findings were also consistent with our prior observation that NMES preferentially strengthened the contralesional motor cortex associated with motor execution, whereas FVS was particularly effective in eliciting transient involuntary attention in somatosensory processing [14]. This demonstrates that repeated NMES and FVS applications induced plasticity similar to their immediate neuromodulatory effects.

It was also noted that cutaneous sensitivity in the median and ulnar nerve territories improved significantly, while ascending dCMC from the FX muscles also increased following training. Although these outcomes were assessed independently, their parallel improvement indicates that FVS contributed to enhanced proprioceptive feedback through the recruitment of cutaneous afferents, which in turn supported the strengthening of ascending somatosensory pathways to the cortex. The repeated EMG-driven activation ensured precise temporal coupling of VME with somatosensory input, promoting neuroplastic changes that ameliorated maladaptive muscle discoordination. Collectively, these findings indicate that the rehabilitative effects of SMI training were mediated not only by improved motor output but also by strengthened sensory integration, with FVS playing a key role in augmenting proprioceptive signaling from spastic flexors.

### Limitations and future work

This study acknowledges several limitations and future directions that require consideration. First, while demonstrating feasibility and rehabilitative efficacy, the EMG-driven EVF-robot requires extensive comparison across the therapeutic effects by the individual contributions of NMES, FVS, and robotic assistance, as well as the comparison with routine care. These comparisons will be carried out in future randomized controlled trials with larger sample sizes, extended intervention durations, and patient stratifications based on chronicity and initial impairment level. Meanwhile, future studies on self-help rehabilitation for chronic stroke in home environments by the EVF-robot will be performed to alleviate the shortfalls of current long-term rehabilitation services for outpatients [36].

Second, the neurophysiological analyses in this study focused on CMC/dCMC and IMC to quantify functional coupling between the cortex and target muscles during rehabilitation. Cortical-level evidence would further strengthen the interpretation of SMI. Poststroke cortical compensation involves widespread network reorganization beyond the sensorimotor cortex [1]. Whole-brain EEG recordings combined with source-level analyses, such as spectral power mapping and network connectivity metrics, could reveal distributed cortical reorganization [75]. Future investigations employing whole-brain EEG during identical training tasks, together with source reconstruction and network connectivity analyses, are expected to clarify whether the SMI facilitated by EMG-driven EVF-robot training is accompanied by broader cortical reorganization and redistribution of inter-areal connectivity.

Third, future research should investigate more sophisticated adaptive frameworks. Specifically, integrating artificial-intelligence-based algorithms could enable real-time adjustment of NMES and FVS parameters based on patient performance indicators such as EMG activity, task success rates, and fatigue levels. This closed-loop adaptive control approach has been increasingly adopted in robotic rehabilitation and brain–computer interface systems, offering the potential for more personalized stimulation protocols, reduced therapist burden, and improved training efficiency [76,77]. Implementing such adaptive algorithms into the EMG-driven EVF-robot system represents a promising avenue for enhancing SMI and promoting long-term functional recovery.

## Conclusion

In this study, we developed an EMG-driven EVF-robot system that enhances SMI in poststroke W/H rehabilitation through targeted somatosensory priming of muscles. The clinical trial provided preliminary evidence that this intervention may modulate compensatory neuroplasticity and enhance neural signals associated with improved coordination of motor control in the distal joints of individuals with chronic stroke. Corticomuscular analyses via EEG and EMG revealed that these improvements stemmed from stronger contralateral cortical control achieved through NMES-robot-assisted SMI in the W/H extensors, and enhanced ascending somatosensory feedback mediated by the FVS to the flexors.

## Data Availability

The data that support the findings of this study are available from the corresponding author upon reasonable request.

## Supplementary Materials

Supplementary 1 Supplementary Text Tables S1 to S5 References [23,36,37,41,78]
