# Supplementary material for: Sensorimotor Integration by Targeted Priming in Muscles with Electromyography-Driven Electro-vibro-feedback in Robot-Assisted Wrist/Hand Rehabilitation after Stroke
Source: Cyborg Bionic Syst. 2026 Jan 27;7:0507. doi: 10.34133/cbsystems.0507 (PMC12835495; doi:10.34133/cbsystems.0507)
Supplement: Supplementary 1 — Supplementary Text Tables S1 to S5 References [23,36,37,41,78] [file cbsystems.0507.f1.docx]

SUPPLEMENTARY MATERIALS

Participant recruitment

After obtaining ethical approval, 15 participants with chronic stroke were screened using the following inclusion criteria: (1) At least 12 months after the onset of a unilateral brain lesion caused by stroke; (2) Absence of visual deficits, and sufficient cognition to follow experimental instructions (Mini-Mental State Examination (MMSE) score > 23) [[78]](file:///\\phd.polyu.edu.hk\home\htxlhu\Supervision\林乐庚\EVF%20ENMS%20paper\revision\final%20submission\The#_CTVL00124d6dd3c9f6d4682a89ca6f57d984afb); (3) Moderate to severe motor disability in the affected UL (15 < FMA < 45) [[37]](file:///\\phd.polyu.edu.hk\home\htxlhu\Supervision\林乐庚\EVF%20ENMS%20paper\revision\final%20submission\Fugl-Meyer#_CTVL001375cb11cdf264b3b877c9054fb69f432); (4) Spasticity score ≤ 3 at the wrist and fingers, assessed using the MAS [[41]](file:///\\phd.polyu.edu.hk\home\htxlhu\Supervision\林乐庚\EVF%20ENMS%20paper\revision\final%20submission\Interrater#_CTVL00192e9ac0bc1634356ad5e3ab90d9b6729); (5) Detectable voluntary EMG signals from the EX and FX muscles on the affected arm (at least three times SD above baseline) [[36]](file:///\\phd.polyu.edu.hk\home\htxlhu\Supervision\林乐庚\EVF%20ENMS%20paper\revision\final%20submission\Home-based#_CTVL001a2b731fe7da44be38bc33dec64d527bf); (6) Passive ROM in the wrist from 45° extension to 60° flexion, and ability to passively extend the metacarpophalangeal (MCP) joints of the fingers to 170° [[23]](file:///\\phd.polyu.edu.hk\home\htxlhu\Supervision\林乐庚\EVF%20ENMS%20paper\revision\final%20submission\An#_CTVL0018efc597c40654ddb88b77d624752031e); (7) No other neurological impairments except those caused by stroke; and (8) Right-handedness prior to stroke onset. Exclusion criteria for stroke participants included (1) poststroke pain, (2) epilepsy, (3) cerebral implantation, (4) pacemaker implantation, and (5) participation in any additional structured rehabilitation programs or experimental interventions during the 7‑week training period or the 3MFU. Written informed consent was obtained from all participants before evaluation, and they were clearly informed that no definite therapeutic benefits could be guaranteed to minimize potential expectancy effects.

Configuration of the system for participants in the SMI training

Before the training program started, configuration parameters of the EMG-driven EVF-robot (Table S1) were individually calibrated based on each participant’s impairment of the W/H joints. The resting baseline EMG ($b_{i}$) was measured during a 5-s muscle relaxation period. The MVC for EX/FX was determined through three 5-s isometric contractions against manual resistance. The activation threshold $\tau_{i}$ was set to 10% MVC above baseline $b_{i}$ to ensure sensitive triggering. NMES pulse width was progressively added from 0 μs until wrist extension reached at least 20°, as required for grasp preparation. The required NMES intensity was 18.29 ± 12.19 μs (mean ± SD), within the adjustable pulse width range (0–300 µs). The FVS amplitude (5.1 G) was confirmed for each participant via oral feedback to elicit somatosensation in FX without discomfort. The inner pressure of pneumatic fingers was adjusted to assist approximately 170° MCP joint flexion at 88.54 ± 4.81 kPa (mean ± SD) remaining within safe levels (<100 kPa). These individually calibrated parameters remained constant for each participant throughout the subsequent SMI training program until recalibration was required to ensure proper system functionality.

**Table S1.** Configuration parameters of the EMG-driven EVF-robot for the recruited 15 participants.

| **Configuration** | **Parameter** | **Ratio** |
| --- | --- | --- |
| EMG threshold for sensitive EMG detection | 10% MVC above the EMG baseline | 15/15 |
| NMES intensity for EX contraction to extend wrist at least 20° | Mean ± SD = 18.29 ± 12.19 µs | 15/15 |
| FVS intensity for perceivable sensation over FX | Nominal amplitude of 5.1 G | 15/15 |
| Inflation of pneumatic muscles to assist ROM of MCP finger joints to around 170° | Mean ± SD = 92.54 ± 4.81 kPa | 15/15 |

**Table S2.** Clinical assessments across evaluation time points.

| **Clinical assessments** | **Pre1** | **Pre2** | **Pre3** | **Post** | **3MFU** | **Δ**  **(Post−Pre)** | **Δ**  **(3MFU−Pre)** | **One-way repeated measures ANOVA** |
| --- | --- | --- | --- | --- | --- | --- | --- | --- |
|  | **Mean value (95% confidence interval)** | | | | | | | ***p***  ***(Partial η^2^)*** |
| FMA-UE | 33.07  (27.26 ~ 38.87) | 33.00  (27.57 ~ 38.43) | 33.20  (28.10 ~ 38.30) | 35.93  (31.01 ~ 40.86) | 35.67  (30.55 ~ 40.79) | 2.84  (4.61 ~ 1.08) | 2.58  (5.00 ~ 0.15) | 0.004** (0.347) |
| FMA-shoulder/elbow | 22.20  (18.43 ~ 25.97) | 22.20  (18.41 ~ 25.99) | 22.40  (18.81 ~ 25.99) | 23.67  (20.58 ~ 26.75) | 23.20  (19.59 ~ 26.81) | 1.40  (2.78 ~ 0.02) | 0.93  (-0.13 ~ 2.00) | 0.045* (0.211) |
| FMA-wrist/hand | 10.87  (8.35 ~ 13.39) | 10.80  (8.61 ~ 12.99) | 10.80  (8.68 ~ 12.92) | 12.27  (9.96 ~ 14.57) | 12.47  (10.35 ~ 14.58) | 1.44  (2.77 ~ 0.12) | 1.64  (0.10 ~ 3.19) | 0.014* (0.267) |
| ARAT-Total | 19.33  (16.18 ~ 22.49) | 21.40  (17.84 ~ 24.96) | 20.80  (17.28 ~ 24.32) | 23.67  (20.97 ~ 26.36) | 23.40  (20.70 ~ 26.10) | 3.16  (0.02 ~ 6.29) | 2.89  (0.61 ~ 5.17) | 0.010** (0.327) |
| ARAT-Grasp | 7.73  (6.09 ~ 9.37) | 8.93  (7.32 ~ 10.55) | 8.33  (6.55 ~ 10.12) | 9.73  (8.54 ~ 10.93) | 9.13  (7.78 ~ 10.49) | 1.40  (2.78 ~ 0.02) | 0.80  (-0.57 ~ 2.18) | 0.031* (0.231) |
| ARAT-Grip | 6.07  (5.33 ~ 6.81) | 6.73  (6.20 ~ 7.27) | 6.33  (5.43 ~ 7.24) | 6.87  (6.32 ~ 7.42) | 6.47  (5.88 ~ 7.05) | 0.49  (0.93 ~ 0.05) | 0.09  (-0.57 ~ 0.75) | 0.119 (0.149) |
| ARAT-Pinch | 1.53  (0.21 ~ 2.86) | 1.93  (0.46 ~ 3.41) | 2.20  (0.96 ~ 3.44) | 3.00  (1.54 ~ 4.47) | 3.67  (2.00 ~ 5.34) | 1.11  (–0.76 ~ 2.99) | 1.78  (0.01 ~ 3.55) | 0.038* (0.219) |
| ARAT-Gross | 4.00  (3.37 ~ 4.63) | 3.80  (2.96 ~ 4.64) | 3.93  (3.36 ~ 4.51) | 4.07  (3.58 ~ 4.56) | 4.13  (3.59 ~ 4.68) | 0.16  (–0.41 ~ 0.72) | 0.22  (-0.21 ~ 0.65) | 0.479 (0.050) |

**Table S3.** Monofilament test scores across evaluation time points.

| **Sensory**  **assessment** | **Pre** | **Post** | **3MFU** | **Δ**  **(Post−Pre)** | **Δ**  **(3MFU−Pre)** | **One-way repeated measures ANOVA** |
| --- | --- | --- | --- | --- | --- | --- |
|  | **Mean value (95% confidence interval)** | | | | | ***p (Partial η^2^)*** |
| Monofilament EX | 3.46  (2.57～4.35) | 2.94  (2.01～3.87) | 3.01  (1.99～4.04) | -0.52  (-1.75 ~ 0.71) | -0.45  (-1.65 ~ 0.76) | 0.521(0.045) |
| Monofilament FX | 3.65  (2.85～4.46) | 3.01  (2.16～3.86) | 3.17  (2.21～4.12) | -0.64  (-1.80 ~ 0.51) | -0.49  (-1.62 ~ 0.64) | 0.272 (0.089) |
| Monofilament 1 | 4.66  (3.80～5.52) | 3.629  (2.71～4.55) | 3.66±1.67  (2.74～4.59) | -1.03  (-2.31 ~ 0.24) | -1.00  (-2.17 ~ 0.17) | 0.043* (0.221) |
| Monofilament 2 | 4.72  (3.96～5.48) | 3.48  (2.66～4.31) | 3.40  (2.49～4.3) | -1.24  (-2.33 ~ -0.16) | -1.33  (-2.24 ~ -0.41) | 0.002** (0.360) |
| Monofilament 3 | 4.80  (3.94～5.65) | 3.69  (2.76～4.62) | 3.68  (2.67～4.69) | -1.11  (-2.39 ~ 0.17) | -1.11  (-2.28 ~ 0.05) | 0.026* (0.253) |
| Monofilament 4 | 4.78  (4.02～5.55) | 3.46  (2.64～4.28) | 3.55  (2.72～4.38) | -1.32  (-2.47 ~ -0.18) | -1.23  (-2.13 ~ -0.33) | 0.003** (0.350) |
| Monofilament 5 | 4.12  (3.10～5.14) | 2.93  (2.10～3.76) | 3.17  (2.13～4.20) | -1.19  (-2.50 ~ 0.12) | -0.95  (-2.01 ~ 0.11) | 0.050 (0.198) |
| Monofilament 6 | 3.72  (2.72～4.72) | 2.88  (2.10～3.66) | 3.11  (2.15 ~ 4.08) | -0.84  (-2.21 ~ 0.52) | -0.61  (-1.58 ~ 0.36) | 0.193 (0.113) |

**Table S4.** MAS scores across evaluation time points.

| **MAS**  **scores** | **Pre1** | **Pre2** | **Pre3** | **Post** | **3MFU** | **Δ**  **(Post−Pre)** | **Δ**  **(3MFU−Pre)** | **Friedman test** |
| --- | --- | --- | --- | --- | --- | --- | --- | --- |
|  | **Medians (IQR)** | | | | | **Rank sum diff** | | ***p***  ***(Kendall’s W)*** |
| MAS-elbow | 1.5 (1～1.5) | 1.5 (1～1.5) | 1.5 (1～2) | 1.5 (1～2) | 1 (0～1.5) | -3 | -12 | 0.029* (0.236) |
| MAS-wrist | 1 (0～1.5) | 1 (0～1.5) | 1 (0～1.5) | 1 (0～1) | 0 (0～1) | -7 | -9.5 | 0.053 (0.196) |
| MAS-finger | 1.5 (1～1.5) | 1 (1～2) | 1.5 (1～2) | 1 (1～1.5) | 1 (1～1.5) | -4.5 | -3 | 0.575 (0.037) |

**Table S5.** Stratified statistical comparisons of post-training FMA-UE improvement by stroke onset time and initial impairment severity.

| **Characteristics** | **Post-training Δ FMA-UE** | **Mann-Whitney U test** |
| --- | --- | --- |
|  | Mean ± SD | *p* (rank-biserial correlation) |
| Stratified by stroke onset time | | |
| ＜5 years (n=9) | 1.89 ± 2.26 | 0.22 (-0.41) |
| ≥5 years (n=6) | 4.28 ± 2.62 |  |
| Stratified by initial impairment severity | | |
| 15＜FMA-UE≤30 (n=7) | 4.14 ± 2.02 | 0.078 (-0.55) |
| 30＜FMA-UE＜45 (n=8) | 1.71 ± 2.65 |  |

REFERENCES

[1] T. A. Jones, “Motor compensation and its effects on neural reorganization after stroke,” Nat. Rev. Neurosci., vol. 18, no. 5, pp. 267–280, 2017.

[2] M. F. Levin, J. A. Kleim, and S. L. Wolf, “What do motor "recovery" and "compensation" mean in patients following stroke?,” Neurorehabil. Neural. Repair., vol. 23, no. 4, pp. 313–319, 2009.

[3] S. Zhou, Z. Guo, and K. Wong *et al.,* “Pathway-specific cortico-muscular coherence in proximal-to-distal compensation during fine motor control of finger extension after stroke,” J. Neural Eng., vol. 18, p. 56034, 2021.

[4] P. Raghavan, “Upper Limb Motor Impairment After Stroke,” Phys. Med. Rehabil. Clin. N. Am., vol. 26, no. 4, pp. 599–610, 2015.

[5] Z. Guo, Q. Qian, and K. Wong *et al.,* “Altered Corticomuscular Coherence (CMCoh) Pattern in the Upper Limb During Finger Movements After Stroke,” Front. Neurol., vol. 11, p. 410, 2020.

[6] N. S. Ward, M. M. Brown, A. J. Thompson, and R. S. J. Frackowiak, “Neural correlates of motor recovery after stroke: a longitudinal fMRI study,” Brain, vol. 126, Pt 11, pp. 2476–2496, 2003.

[7] C. Delcamp, C. Cormier, A. Chalard, D. Amarantini, and D. Gasq, “Changes in intermuscular connectivity during active elbow extension reveal a functional simplification of motor control after stroke,” Front. Neurosci., vol. 16, p. 940907, 2022.

[8] N. Takeuchi and S. Izumi, “Maladaptive plasticity for motor recovery after stroke: mechanisms and approaches,” Neural Plast., vol. 2012, no. 1, p. 359728, 2012.

[9] X. Hu, K. Tong, V. S. Tsang, and R. Song, “Joint-angle-dependent neuromuscular dysfunctions at the wrist in persons after stroke,” Arch. Phys. Med. Rehabil., vol. 87, no. 5, pp. 671–679, 2006.

[10] A. Biasiucci, R. Leeb, and I. Iturrate *et al.,* “Brain-actuated functional electrical stimulation elicits lasting arm motor recovery after stroke,” Nat. Commun., vol. 9, no. 1, p. 2421, 2018.

[11] S. H. Frey, L. Fogassi, and S. Grafton *et al.,* “Neurological principles and rehabilitation of action disorders: computation, anatomy, and physiology (CAP) model,” Neurorehabil. Neural. Repair., vol. 25, 5 Suppl, 6S-20S, 2011.

[12] A. S. Asan, J. R. McIntosh, and J. B. Carmel, “Targeting Sensory and Motor Integration for Recovery of Movement After CNS Injury,” Front. Neurosci., vol. 15, p. 791824, 2021.

[13] L. Lin, W. Qing, and Z. Zheng *et al.,* “Somatosensory integration in robot-assisted motor restoration post-stroke,” Front. Aging. Neurosci., vol. 16, 2024.

[14] L. Lin, W. Qing, and Y. Huang *et al.,* “Comparison of Immediate Neuromodulatory Effects between Focal Vibratory and Electrical Sensory Stimulations after Stroke,” Bioengineering, vol. 11, no. 3, p. 286, 2024.

[15] Q. Qian, C. Nam, and Z. Guo *et al.,* “Distal versus proximal - an investigation on different supportive strategies by robots for upper limb rehabilitation after stroke: a randomized controlled trial,” J. NeuroEng. Rehabil., vol. 16, 2019.

[16] F. Zhang, L. Lin, L. Yang, and Y. Fu, “Variable impedance control of finger exoskeleton for hand rehabilitation following stroke,” Ind. Robot, vol. 47, no. 1, pp. 23–32, 2019.

[17] V. Klamroth-Marganska, J. Blanco, and K. Campen *et al.,* “Three-dimensional, task-specific robot therapy of the arm after stroke: a multicentre, parallel-group randomised trial,” Lancet Neurol., vol. 13, no. 2, pp. 159–166, 2014.

[18] J. Mehrholz, M. Pohl, T. Platz, J. Kugler, and B. Elsner, “Electromechanical and robot-assisted arm training for improving activities of daily living, arm function, and arm muscle strength after stroke,” Cochrane Database Syst. Rev., vol. 9, no. 9, CD006876, 2018.

[19] K. Hu, Z. Ma, S. Zou, J. Li, and H. Ding, “Impedance Sliding-Mode Control Based on Stiffness Scheduling for Rehabilitation Robot Systems,” Cyborg. Bionic Syst., vol. 5, p. 99, 2024.

[20] L. Lin, F. Zhang, L. Yang, and Y. Fu, “Design and modeling of a hybrid soft-rigid hand exoskeleton for poststroke rehabilitation,” Int. J. Mech. Sci., vol. 212, p. 106831, 2021.

[21] F. Zhang, L. Lin, L. Yang, and Y. Fu, “Design of an Active and Passive Control System of Hand Exoskeleton for Rehabilitation,” Appl. Sci.-Basel, vol. 9, no. 11, p. 2291, 2019.

[22] X. Hu, K. Tong, R. Song, X. J. Zheng, and W. F. W. Leung, “A comparison between electromyography-driven robot and passive motion device on wrist rehabilitation for chronic stroke,” Neurorehabil. Neural. Repair., vol. 23, no. 8, pp. 837–846, 2009.

[23] C. Nam, W. Rong, and W. Li *et al.,* “An Exoneuromusculoskeleton for Self-Help Upper Limb Rehabilitation After Stroke,” Soft Robot., 2020.

[24] M. E. Stoykov and S. Madhavan, “Motor priming in neurorehabilitation,” J. Neurol. Phys. Ther., vol. 39, no. 1, pp. 33–42, 2015.

[25] M. E. Stoykov, C. Heidle, S. Kang, L. Lodesky, L. E. Maccary, and S. Madhavan, “Sensory-Based Priming for Upper Extremity Hemiparesis After Stroke: A Scoping Review,” OTJR-Occup. Particip. Health, 15394492211032606, 2021.

[26] Y. Huang, C. Nam, and W. Li *et al.,* “A comparison of the rehabilitation effectiveness of neuromuscular electrical stimulation robotic hand training and pure robotic hand training after stroke: A randomized controlled trial,” Biomed. Signal Process. Control, vol. 56, p. 101723, 2020.

[27] A. Insausti-Delgado, E. López-Larraz, J. Omedes, and A. Ramos-Murguialday, “Intensity and Dose of Neuromuscular Electrical Stimulation Influence Sensorimotor Cortical Excitability,” Front. Neurosci., vol. 14, p. 593360, 2020.

[28] G. M. Graham, T. A. Thrasher, and M. R. Popovic, “The effect of random modulation of functional electrical stimulation parameters on muscle fatigue,” IEEE Trans. Neural Syst. Rehabil. Eng., vol. 14, no. 1, pp. 38–45, 2006.

[29] R. Souron, T. Besson, G. Y. Millet, and T. Lapole, “Acute and chronic neuromuscular adaptations to local vibration training,” Eur. J. Appl. Physiol., vol. 117, no. 10, pp. 1939–1964, 2017.

[30] D. Zeng, W. Lei, and Y. Kong *et al.,* “Effects of vibration therapy for post-stroke spasticity: a systematic review and meta-analysis of randomized controlled trials,” Biomed. Eng. Online, vol. 22, no. 1, p. 121, 2023.

[31] Y. Lee, K. Lin, H. Cheng, C. Wu, Y. Hsieh, and C. Chen, “Effects of combining robot-assisted therapy with neuromuscular electrical stimulation on motor impairment, motor and daily function, and quality of life in patients with chronic stroke: a double-blinded randomized controlled trial,” J. NeuroEng. Rehabil., vol. 12, p. 96, 2015.

[32] C. R. Carvalho, J. M. Fernández, A. J. Del-Ama, F. Oliveira Barroso, and J. C. Moreno, “Review of electromyography onset detection methods for real-time control of robotic exoskeletons,” J. NeuroEng. Rehabil., vol. 20, no. 1, p. 141, 2023.

[33] Y. Huang, W. P. Lai, Q. Qian, X. Hu, E. W. C. Tam, and Y. Zheng, “Translation of robot-assisted rehabilitation to clinical service: a comparison of the rehabilitation effectiveness of EMG-driven robot hand assisted upper limb training in practical clinical service and in clinical trial with laboratory configuration for chronic stroke,” Biomed. Eng. Online, vol. 17, no. 1, p. 91, 2018.

[34] C. Nam, W. Rong, W. Li, Y. Xie, X. Hu, and Y. Zheng, “The Effects of Upper-Limb Training Assisted with an Electromyography-Driven Neuromuscular Electrical Stimulation Robotic Hand on Chronic Stroke,” Front. Neurol., vol. 8, p. 679, 2017.

[35] X. Lou, S. Xiao, Y. Qi, X. Hu, Y. Wang, and X. Zheng, “Corticomuscular coherence analysis on hand movement distinction for active rehabilitation,” Comput. Math. Method Med., vol. 2013, p. 908591, 2013.

[36] C. Nam, B. Zhang, and T. Chow *et al.,* “Home-based self-help telerehabilitation of the upper limb assisted by an electromyography-driven wrist/hand exoneuromusculoskeleton after stroke,” J. NeuroEng. Rehabil., vol. 18, no. 1, p. 137, 2021.

[37] K. J. Sullivan, J. K. Tilson, and S. Y. Cen *et al.,* “Fugl-Meyer assessment of sensorimotor function after stroke: standardized training procedure for clinical practice and clinical trials,” Stroke, vol. 42, no. 2, pp. 427–432, 2011.

[38] D. Carrol, “A quantitative test of upper extremity function,” J. Chronic Dis., vol. 18, pp. 479–491, 1965.

[39] M. Suda, M. Kawakami, and K. Okuyama *et al.,* “Validity and Reliability of the Semmes-Weinstein Monofilament Test and the Thumb Localizing Test in Patients With Stroke,” Front. Neurol., vol. 11, p. 625917, 2020.

[40] J. L. Bowden, G. G. Lin, and P. A. McNulty, “The prevalence and magnitude of impaired cutaneous sensation across the hand in the chronic period post-stroke,” PLoS One, vol. 9, no. 8, e104153, 2014.

[41] R. W. Bohannon and M. B. Smith, “Interrater reliability of a modified Ashworth scale of muscle spasticity,” Phys. Ther., vol. 67, no. 2, pp. 206–207, 1987.

[42] M. Houston, G. Seo, and F. Fang *et al.,* “Modulating Inter-Muscular Coordination Patterns in the Upper Extremity Induces Changes to Inter-Muscular, Cortico-Muscular, and Cortico-Cortical Connectivity,” IEEE J. Biomed. Health Inform., PP, 2024.

[43] S. Bao, W. Leung, V. C. K Cheung, P. Zhou, and K. Tong, “Pathway-specific modulatory effects of neuromuscular electrical stimulation during pedaling in chronic stroke survivors,” J. NeuroEng. Rehabil., vol. 16, no. 1, p. 143, 2019.

[44] M. Seeck, L. Koessler, and T. Bast *et al.,* “The standardized EEG electrode array of the IFCN,” Clin. Neurophysiol., vol. 128, no. 10, pp. 2070–2077, 2017.

[45] T. Jia, J. Sun, C. McGeady, L. Ji, and C. Li, “Enhancing Brain-Computer Interface Performance by Incorporating Brain-to-Brain Coupling,” Cyborg. Bionic Syst., vol. 5, p. 116, 2024.

[46] H. Wen, Y. Zhong, L. Yao, and Y. Wang, “Neural Correlates of Motor/Tactile Imagery and Tactile Sensation in a BCI paradigm: A High-Density EEG Source Imaging Study,” Cyborg. Bionic Syst., vol. 5, p. 118, 2024.

[47] R. Oostenveld, P. Fries, E. Maris, and J. Schoffelen, “FieldTrip: Open source software for advanced analysis of MEG, EEG, and invasive electrophysiological data,” Comput. Intell. Neurosci., vol. 2011, p. 156869, 2011.

[48] A. Delorme and S. Makeig, “EEGLAB: an open source toolbox for analysis of single-trial EEG dynamics including independent component analysis,” J. Neurosci. Methods, vol. 134, no. 1, pp. 9–21, 2004.

[49] M. Houston, X. Li, P. Zhou, S. Li, J. Roh, and Y. Zhang, “Alterations in Muscle Networks in the Upper Extremity of Chronic Stroke Survivors,” IEEE Trans. Neural Syst. Rehabil. Eng., vol. 29, pp. 1026–1034, 2021.

[50] J. Zhang, M. Wang, M. Alam, Y. Zheng, F. Ye, and X. Hu, “Effects of non-invasive cervical spinal cord neuromodulation by trans-spinal electrical stimulation on cortico-muscular descending patterns in upper extremity of chronic stroke,” Front. Bioeng. Biotechnol., vol. 12, p. 1372158, 2024.

[51] K. B. Wilkins, M. Owen, C. Ingo, C. Carmona, J. P. A. Dewald, and J. Yao, “Neural Plasticity in Moderate to Severe Chronic Stroke Following a Device-Assisted Task-Specific Arm/Hand Intervention,” Front. Neurol., vol. 8, p. 284, 2017.

[52] A. Brovelli, M. Ding, A. Ledberg, Y. Chen, R. Nakamura, and S. L. Bressler, “Beta oscillations in a large-scale sensorimotor cortical network: directional influences revealed by Granger causality,” Proc. Natl. Acad. Sci. U. S. A., vol. 101, no. 26, pp. 9849–9854, 2004.

[53] A. Delorme, T. Mullen, and C. Kothe *et al.,* “EEGLAB, SIFT, NFT, BCILAB, and ERICA: New Tools for Advanced EEG Processing,” Comput. Intell. Neurosci., vol. 2011, pp. 1–12, 2011.

[54] J. Geweke, “Measurement of Linear Dependence and Feedback between Multiple Time Series,” J. Am. Stat. Assoc., vol. 77, no. 378, pp. 304–313, 1982.

[55] C. L. Witham, M. Wang, and S. N. Baker, “Corticomuscular coherence between motor cortex, somatosensory areas and forearm muscles in the monkey,” Front. Syst. Neurosci., vol. 4, 2010.

[56] F. Babiloni, F. Cincotti, and C. Babiloni *et al.,* “Estimation of the cortical functional connectivity with the multimodal integration of high-resolution EEG and fMRI data by directed transfer function,” NeuroImage, vol. 24, no. 1, pp. 118–131, 2005.

[57] F. Giorgi, D. Donati, D. Platano, and R. Tedeschi, “Focal Vibration Therapy for Motor Deficits and Spasticity Management in Post-Stroke Rehabilitation,” Brain Sci., vol. 14, no. 11, 2024.

[58] K. Li, Y. Sun, J. Li, H. Li, J. Zhang, and L. Wang, “Characterization of Muscle Fatigue Degree in Cyclical Movements Based on the High-Frequency Components of sEMG,” Biomimetics (Basel, Switzerland), vol. 10, no. 5, 2025.

[59] C. Basla, L. Chee, and G. Valle *et al.,* “Sensory-Motor Neurostimulation to Enhance Exosuit Performance,” in *2023 International Conference on Rehabilitation Robotics (ICORR)*, Singapore, 2023, pp. 1–6.

[60] C. Gao, Y. Chen, and Y. Wei *et al.,* “Combined Immersive and Nonimmersive Virtual Reality With Mirror Therapy for Patients With Stroke: Systematic Review and Meta-Analysis of Randomized Controlled Trials,” J. Med. Internet Res., vol. 27, e73142, 2025.

[61] M. Goyal, R. McDonough, M. Fisher, and J. Ospel, “The Challenge of Designing Stroke Trials That Change Practice: MCID vs. Sample Size and Pragmatism,” J. Stroke, vol. 24, no. 1, pp. 49–56, 2022.

[62] X. Li, Y. He, D. Wang, and M. J. Rezaei, “Stroke rehabilitation: from diagnosis to therapy,” Front. Neurol., Volume 15 - 2024, 2024.

[63] S. J. Page, G. D. Fulk, and P. Boyne, “Clinically important differences for the upper-extremity Fugl-Meyer Scale in people with minimal to moderate impairment due to chronic stroke,” Phys. Ther., vol. 92, no. 6, pp. 791–798, 2012.

[64] J. H. van der Lee, V. de Groot, H. Beckerman, R. C. Wagenaar, G. J. Lankhorst, and L. M. Bouter, “The intra- and interrater reliability of the action research arm test: a practical test of upper extremity function in patients with stroke,” Arch. Phys. Med. Rehabil., vol. 82, no. 1, pp. 14–19, 2001.

[65] J. Cohen, *Statistical Power Analysis for the Behavioral Sciences*. Hoboken: Taylor and Francis, 2013.

[66] L. Rocchi, A. Suppa, and G. Leodori *et al.,* “Plasticity Induced in the Human Spinal Cord by Focal Muscle Vibration,” Front. Neurol., vol. 9, p. 935, 2018.

[67] S. Pizzamiglio, M. de Lillo, U. Naeem, H. Abdalla, and D. L. Turner, “High-Frequency Intermuscular Coherence between Arm Muscles during Robot-Mediated Motor Adaptation,” Front. Physiol., vol. 7, p. 668, 2016.

[68] J. H. Park, J. H. Shin, H. Lee, J. Roh, and H. S. Park, “Alterations in intermuscular coordination underlying isokinetic exercise after a stroke and their implications on neurorehabilitation,” J. NeuroEng. Rehabil., vol. 18, no. 1, p. 110, 2021.

[69] J. R. Carey, T. J. Kimberley, and S. M. Lewis *et al.,* “Analysis of fMRI and finger tracking training in subjects with chronic stroke,” Brain, vol. 125, Pt 4, pp. 773–788, 2002.

[70] R. Kristeva, L. Patino, and W. Omlor, “Beta-range cortical motor spectral power and corticomuscular coherence as a mechanism for effective corticospinal interaction during steady-state motor output,” NeuroImage, vol. 36, no. 3, pp. 785–792, 2007.

[71] P. Cordo, S. Wolf, and J. Lou *et al.,* “Treatment of severe hand impairment following stroke by combining assisted movement, muscle vibration, and biofeedback,” J. Neurol. Phys. Ther., vol. 37, no. 4, pp. 194–203, 2013.

[72] A. Viganò, C. Celletti, and G. Giuliani *et al.,* “Focal Muscle Vibration (fMV) for Post-Stroke Motor Recovery: Multisite Neuroplasticity Induction, Timing of Intervention, Clinical Approaches, and Prospects from a Narrative Review,” Vibration, vol. 6, no. 3, pp. 645–658, 2023.

[73] W. Y. Tseng, I. H. Tseng, and L. W. Chou, “The Effects of Sensory Electrical Stimulation and Local Vibration on Motor Learning and Motor Function,” Phys. Ther. Res., vol. 28, no. 1, pp. 9–13, 2025.

[74] P. J. Cordo, J. L. Horn, D. Künster, A. Cherry, A. Bratt, and V. Gurfinkel, “Contributions of skin and muscle afferent input to movement sense in the human hand,” J. Neurophysiol., vol. 105, no. 4, pp. 1879–1888, 2011.

[75] G. Zhan, S. Chen, and Y. Ji *et al.,* “EEG-Based Brain Network Analysis of Chronic Stroke Patients After BCI Rehabilitation Training,” Front. Hum. Neurosci., vol. 16, p. 909610, 2022.

[76] G. Nicora, S. Pe, and G. Santangelo *et al.,* “Systematic review of AI/ML applications in multi-domain robotic rehabilitation: trends, gaps, and future directions,” J. NeuroEng. Rehabil., vol. 22, no. 1, p. 79, 2025.

[77] W. Jin, X. Zhu, and L. Qian *et al.,* “Electroencephalogram-based adaptive closed-loop brain-computer interface in neurorehabilitation: a review,” Front. Comput. Neurosci., Volume 18 - 2024, 2024.

[78] T. N. Tombaugh and N. J. McIntyre, “The mini-mental state examination: a comprehensive review,” J. Am. Geriatr. Soc., vol. 40, no. 9, pp. 922–935, 1992.
